# Cases Reported to the Buffalo Medical Association

**Published:** 1851-06

**Authors:** James S. Hawley

**Affiliations:** Secretary


*Abstract of cases reported to the Buffalo Medical Association.*—Prepared by JAMES S. HAWLEY, Secretary.

May, 6th: *Fracture of the Spine.*—Reported by Dr. VANDEVENTER.—Patient, a negro, aged forty-five years: received the injury from the falling of a ballustrade, striking him edgewise between the shoulders. The immediate effect was a loss of consciousness. After its restoration the patient was found to be deprived of sensibility and motion below the seat of the injury, excepting the arms, which were but partially paralyzed. Consciousness, respiration, and voice remained perfect. Neither the urine nor the fæces passed without artificial aid. The only external signs of injury were the abrasion, and pain upon pressure over the seat of the injury: skin cold, and pulse depressed at first: re-action came on only partially: increased frequency of the pulse and labored, catching, respiration came on in about thirty-six hours, and death occurred forty hours after the injury.

Autopsy eight hours after death. No morbid appearances were found in the substance of the brain,— about an ounce and a half of bloody serum at the base of the brain, and in the theca spinalis,— seventh cervical vetebra fractured,— arches broken down, and the spinous process driven in upon the medulla.

May 20: *Placenta Previa and turning.*— Reported by Dr. Hawley.— Patient, middle-aged, full habit, in her seventh pregnacy. Hemorrhage first appeared about the middle of the eighth month,—was suspended by repose,— reappeared in two weeks very profusely,— arrested by tampon,— was renewed in eighteen hours, with the expulsion of the tampon, which was re-applied without effect. Examination: os uteri sufficiently dilated to receive the ends of all the fingers and thumb,— placenta situated on the inferior posterior portion of the uterus,— one side extending entirely over the mouth, sufficiently detached to allow the hand to pass,— turned without allowing the waters to escape,— uterine contractions came on promptly,— hemorrhage ceased from the time the labor commenced,— child delivered dead in an hour and a half: patient discharged on the fifth day.

